# Factors that hinder medical career aspirations: A nationwide questionnaire survey of teachers in charge of career guidance in Japanese high schools

**DOI:** 10.1371/journal.pone.0270477

**Published:** 2022-06-24

**Authors:** Junji Otaki, Kikuko Taketomi, Machiko Shibahara, Yoko Watanabe, Shizuko Nagata-Kobayashi, Yoshimi Harada, Hiroshi Mitoma

**Affiliations:** 1 Center for Medical Education and International Relations, Faculty of Medicine, Hokkaido University, Sapporo, Hokkaido, Japan; 2 Department of General Medicine and Primary Care, Tokyo Medical University, Shinjuku-ku, Tokyo, Japan; 3 Medical Education Center, Faculty of Medicine, Kyoto University, Kyoto, Japan; 4 Chair of Lifelong Education, Graduate School of Education, Kyoto University, Kyoto, Japan; 5 Department of Medical Education, Tokyo Medical University, Shinjuku-ku, Tokyo, Japan; University of Eastern Finland: Ita-Suomen yliopisto, FINLAND

## Abstract

Despite concerns raised on the inequality in healthcare provision in Japan, little is known about the factors that hinder candidates’ application to medical schools. A nationwide cross-sectional survey was conducted to identify the impact of economic factors and living place on students’ choice of and preparation for medical school. The survey was administered to high school teachers with career advisory roles, as they support and likely influence students’ choice and decision on this matter. Responses totaling 1,094 were obtained from 1,746 high schools across Japan. The ratio of high schools with two or more students enrolled in medical schools every year is higher in private schools, those with high tuition, and those located in big cities. Approximately 66.8% of the respondents agreed that "It is difficult for students in economically disadvantaged families to enroll in medical schools;" 42.0% agreed that "Some students gave up on aspiring to enter medical schools because they could not afford it," and 61.2% agreed that "Students living in urban areas are more likely to enroll in medical schools." When asked about the percentage of students attending prep school among those aspiring for a medical career, significantly more respondents from private versus public high schools answered "80% or more." When asked about the percentage of parents who are doctors or dentists among students aspiring for a medical career, significantly more respondents from private versus public high schools answered "50% or more.” The results suggest that students from lower-income families and those living in rural areas are more likely to be disadvantaged when choosing a medical career (because of financial difficulties) than those who live in urban areas and come from wealthier families. The results imply that economic and geographical divides in medical admission are reflected in high school teachers’ perception of and support provided to students.

## Introduction

Several studies have found that in the United States, a certain group of students enjoy more advantages in their competitive quest to enter medical school [1]. Meanwhile, among factors such as access to role models and advice on careers in medicine in the United Kingdom [2] and the impact of immigration in the United States [3], certain economic backgrounds can lead to significant advantages for students when choosing, preparing for, and enrolling in medical schools [4, 5].

In Japan’s case, despite concerns raised regarding the inequality in healthcare provision, little is known about the economic factors that hinder possible candidates from applying to medical schools, partly because of the difficulty of gathering applicant information. In the admission process, applicants are generally not required to submit any economic background information. This makes it challenging for medical educators and policymakers to grasp the applicants’ economic situation and discuss possible factors that may hinder them from pursuing a medical career, even though it is widely recognized that children from the families of doctors are more likely to be admitted because of their financial advantage.

Based on a survey of parents of Japanese high school students, Kobayashi analyzed and reported on the burden of education costs that make it difficult for students to advance to universities. According to the report, the proportion of those who wished to pursue a career in medicine or dentistry was significantly lower for those in the low-income bracket [6]. However, for this study’s scope, we could not find any other report on the economic factors that influence medical career aspirations.

Understanding economic hindrance is particularly important in a society where private medical schools play a significant role in supplying the future medical workforce. Japan has 81 medical schools, approximately 60% of which are public, and the rest are private. In total, approximately 9,000 students enroll every year. Tuition fees hugely differ between public and private schools. A student at a public medical school is required to spend about 3.5 million yen (about 35,000 US dollars) during their six-year undergraduate education, versus approximately 20 to 50 million yen (about 200,000 to 500,000 US dollars) at a private school.

Many medical schools, both private and public, have set up a “regional quota” that covers part of the tuition fees and, in some cases, annual stipends in return for future work contracts (ranging from six to nine years) in designated areas or specialties where uneven distribution is particularly severe [7]. However, the number of students with this scholarship, which differs from school to school, is limited to almost one-sixth of the total enrollment capacity, and the scholarship amount of most private schools is insufficient to cover the tuition.

The selection procedures also tend to be advantageous for students with higher financial ability. Unlike in other countries, such as the United States, students in Japan do not take a unified examination for medical school admission. Instead, most medical schools, both public and private, conduct their own advanced-level examinations to assess students’ abilities to pursue medical studies, in addition to the Common Test for University Admissions, which is taken by most candidates who wish to attend higher education, regardless of the subjects. This school-specific advanced examination system requires students to exert tremendous effort to prepare for each medical school.

It is highly challenging for students to prepare for specific examinations without access to private tutoring [8]. Some private companies have developed preparation programs specifically for medical school entry that start as early as the age of 12. Tuition fees for such private services could reach up to 6 million yen (approximately 60,000 US dollars) per year. Likewise, some private junior and high schools are keen on preparing their students for medical school as they are allowed to design and deliver curricula more freely than public schools. Such private schools tend to be located in urban areas, contributing to the urban-rural divide. In such an educational environment, a survey might clarify whether hindrances to medical career aspirations could be estimated by the type and location of the high school.

Considering the medical admission system in Japan, students’ economic situation and living place likely play a significant role in their preparation for a medical career and success in the selection process. Although these factors may affect various aspects of the medical school enrollment process, this study particularly focuses on the input of high school teachers, as they are usually the key resource persons for students to rely on, regardless of the students’ residence or economic situation. Understanding how high school teachers perceive and provide support to students will help clarify how economic ability and location influence students’ choice, preparation, and enrollment in medical schools.

## Methods

This study conducted a nationwide cross-sectional questionnaire survey.

As previously mentioned, it is difficult to investigate the financial status and place of residence of examinees and enrollees in medical schools in Japan because accessible information is lacking. Therefore, this study focused on the input of teachers in charge of career guidance in high schools that have a relatively high rate of university advancement. We speculated that they had a detailed understanding of the student’s situation.

When the survey was conducted in 2015, there were 4,925 high schools across Japan, out of which we selected 1,746, based on two articles published in the same year in a major weekly magazine, “Shukan Asahi,” that ranked both private and public high schools in terms of the number of students successfully enrolling in higher education institutions [9, 10]. According to the articles, more than one student at the schools on this list advanced to a well-known university or four-year college. The high schools included in the ranking are well recognized for their achievement and therefore, their schoolteachers are considered to be more experienced in advising and supporting students in their preparation for universities (including medical schools) than teachers from other schools that are not in the ranking. Letters and questionnaires were sent to these 1,746 schools in June 2015, asking a teacher with career-counseling roles to respond.

The questionnaire items (Table 1) were designed to collect objective data on students enrolling in medical schools as well as the respondent’s perceptions as career adviser at school. The questions were developed primarily through our discussions at the International Symposium [11] on Challenges and Reforms of Medical School Entrance Examinations (held on October 26, 2013) and our research group. At the symposium, researchers from Japan, the United Kingdom, the United States, Taiwan, and Canada gathered to discuss how to deal with the harm of excessive academic achievement tests, measures against social disparities, and evaluation methods that are consistent with admission policies. The participants of the study were requested to answer either multiple choice or short answers for most questions. They were also encouraged to leave comments regarding their experience.

**Table 1:** Main contents of the questionnaire.

**Respondent attributes**
Subject for which the respondent is in charge of
Years of experience as a high school teacher^a^
**Attributes of the high school**
Size of school (the number of third-grade students)^b^
Annual average university admission rate^a^
Annual average number of students enrolled in medical schools (private and public)^a^
Tuition fees^a^
Percentage of students aspiring to enter medical school who attend prep school^a^
**Respondents’ perception of entrance exams for medical schools**
Important skills and abilities necessary to become medical students^c^
Impact of family’s economic condition and student’s place of living on career choice^c^
**Attributes of students aspiring to enter a medical school**
Percentage of parents who are doctors or dentists^a^
**Issues the respondent encountered regarding medical school admission** ^d^

^a^Interval scale was used for this item.

^b^Actual count was asked for this item.

^c^Likert scale was used for this item.

^d^Free writing was used for this item.

Participants answered the question regarding perception on a 7-point Likert scale, with the fourth option being neutral (I can’t say either). The three options on the affirmative side (I strongly think so; I think so; If anything, I think so) were considered affirmative answers.

The responses were aggregated and analyzed through descriptive statistics, t-test, F-test, and chi-square analysis. The statistical software JMP ® Pro 16(SAS Institute Inc., Cary, NC, USA) was used for the analysis. This survey was conducted with approval from the Hokkaido University Ethical Committee, which was later deemed unnecessary as there were no applicable ethical considerations as of June 19, 2015.

## Results

Answers were obtained from 1,094 individuals (from 671 public schools and 423 private schools). The response rate was 62.7%. As for the respondents themselves, 1,045 (95.5%) had more than 10 years of experience as teachers, and 1,040 (95.1%) were in charge of one or more subjects other than career guidance and managerial positions.

### (1) Size of schools

The average number of 3^rd^ grade students in each high school was 273.9 ± 122.2 (range: 20–1, 400); 99 or fewer at 43 schools (3.9%); 100–199 at 237 schools (21.7%); 200–299 at 383 schools (35.0%); 300–399 students at 319 schools (29.2%); and more than 400 students at 97 schools (8.9%). The participants from 16 schools (1.5%) did not answer this question. When stratified, the average number of students was 265.7 ± 85.1 in public schools; 287.1 ± 164.6 in private schools; 295.2 ± 124.5 in schools located in cities with a population of 200,000 or more; and 246.2 ± 113.5 in schools located in municipalities with a population of less than 200,000.

### (2) University admission rate and number of students enrolled in medical schools

On an annual average basis, there were 612 high schools (55.9%) with an admission rate of 80% or more to 4-year colleges; 136 schools (12.4%) with 11 or more graduates enrolled in medical schools; 290 schools (26.5%) with 2 to 10 students; and 665 schools (60.8%) with one or fewer students.

### (3) Tuition fees

Table 2 shows the answers regarding tuition fees for the three years of high school. Only 13 out of 423 private schools cost less than one million yen (about $ 10,000). For public schools, only 20 out of 671 respondents answered one million yen or more.

**Table 2:** Relationship between total tuition fees of high schools and their attributes (school type and number of students enrolled in medical schools).

Amount of total tuition for 3 years^a^	All schools	Type of high school		No. of students enrolled in medical schools^b^		
		Public	Private	≧10	2–9	0–1
	n = 1,094	671	423	136	290	665
	(100%)^c^	(100%)^c^	(100%)^c^	(100%)^c^	(100%)^c^	(100%)^c^
Less than 500,000 yen^d^	324	321	3	27	71	224
	(29.6%)	(47.8)	(0.7)	(19.9)	(24.5)	(33.7)
500,000 yen~990,000 yen	210	200	10	26	48	136
	(19.2)	(29.8)	(2.4)	(19.1)	(16.6)	(20.5)
1,000,000 yen~1,990,000 yen	203	19	184	25	56	122
	(18.6)	(2.8)	(43.5)	(18.4)	(19.3)	(18.3)
2,000,000 yen or more	187	1	186	44	65	78
	(17.1)	(0.1)	(44.0)	(32.4)	(22.4)	(11.7)
Do not know or no answer	170	130	40	14	50	105
	(15.5)	(19.4)	(9.5)	(10.3)	(17.2)	(15.8)

^a^The total amount of admission fees, tuition fees, facility equipment fees, and other expenses that need to be paid. The cost of prep school is not included.

^b^Three schools were excluded because of missing data.

^c^Numbers in parentheses indicate percentages for each column.

^d^$1 is approximately100 yen

In high schools where the tuition fee for three years is less than one million yen (approximately $ 10,000), 32.2% (172 out of 534 schools) of the schools had two or more students enrolled in medical schools every year. Meanwhile, in high schools with tuition fees of two million yen (about 20,000 dollars) or more, the ratio was significantly higher (p < 0.001) at 58.3% (109 out of 187 schools).

### (4) Impact of the family’s economic condition on the student’s entry to medical school

Seven hundred thirty-one respondents (66.8% of all participants) answered affirmatively to the statement, "It is difficult for students from economically disadvantaged families to enroll in medical school."

Out of 1,076 respondents, 42.0% (448) answered affirmatively to the statement, "Some students gave up on aspiring to enter a medical school because they could not afford it." Respondents from private schools accounted for 47.5% (201 out of 423) of the affirmative responses, and those from public schools accounted for 36.8% (247 out of 671). Regarding high school tuition fees, respondents from schools with high tuition fees (two million yen (about 20,000 dollars) or more in total for the three years) accounted for 49.7% (93 out of 187) of the affirmative responses. Meanwhile, respondents from schools with low tuition fees (less than one million yen (about $ 10,000)) accounted for 37.8% (202 out of 534) of the affirmative responses.

Table 3 shows this ratio examined according to eight regional categories in Japan. In the Kanto area, including Tokyo, the capital city, the percentage of "some students gave up" was high at 60.0%. It was relatively low in Hokkaido (20.8%), Chugoku-Shikoku (24.1%), and Tohoku (28.0%), and there was a statistically significant difference from the Kanto area (χ2 (7, N = 1,076) = 76.95, p < 0.0001).

**Table 3:** Responses to the statement "some students gave up on aspiring to enter a medical school because they could not afford it," and relevant data compared by regional category.

Regional category	Total no. of respondents	No. and percentage of affirmative responses^a^		Population in 2015 (thousands)	No. of high schools to which the respondents belong		No. of medical schools in 2015^b^
					Type of school	City/town size of location	Public / private
					Public /private	≧200,000 /<200,000	
Hokkaido	72	15	20.8%	5,382	47 / 25	37 / 35	3 / 0
Tohoku	100	28	28.0	8,983	80 / 20	52 / 48	5 / 1
Kanto	300	180	60.0	42,995	139 /161	202 / 98	6 / 17
Hokuriku/Koshinetsu	92	39	42.4	8,245	77 / 15	37 / 55	6 / 1
Chubu^c^	109	45	41.3	13,215	75 / 34	58 / 51	4 / 2
Kinki^d^	173	66	38.1	22,541	93 / 80	108 / 65	9 / 4
Chugoku/Shikoku	108	26	24.1	11,284	69 / 39	47 / 61	9 / 1
Kyushu/Okinawa	122	49	40.2	14,450	80 / 42	68 / 54	8 / 3
Total	1076^e^	448	41.0	127,095	660 / 416	609 / 467	50/ 29

^a^Percentage is the proportion of affirmative responses of respondents by region.

^b^Two medical schools that were newly opened after 2015.

^c^The Chubu area includes Shizuoka, Aichi, and Gifu prefectures in this study.

^d^The Kinki area includes Mie, Shiga, Kyoto, Osaka, Hyogo, Nara, and Wakayama prefectures in this study.

^e^Eighteen schools did not answer this question.

### (5) Impact of the location of the student’s residence on their choice of a medical career

Approximately 61.2% of all respondents (670 out of 1,094) answered affirmatively to the statement "Students living in urban areas are more likely to enroll in medical school."

### (6) Percentage of students attending prep school among those aspiring to enter medical school

Regarding the percentage of students attending prep school among those aspiring to enter medical school (1,063 respondents answered), the number of students that responded "I don’t know" was 428 (39.1%); "80% or more" was 236 (22.2%); "50–79%" was 109 (10.3%); "10–49%" was 64 (6.0%), and "less than 10%" was 226 (21.3%).

For these answers (excluding "I don’t know"), we tested the difference in the percentage of stratified responses. The main results are as follows. The percentage of respondents who answered "80% or more" was significantly (p < 0.0001) higher in private high schools (134 out of 279 or 48.0%) than in public high schools (102 out of 356 or 28.7%). It was also significantly (p < 0.0001) higher in high schools with a population of 200,000 or more (166 out of 373 or 44.5%) than in high schools with a population of less than 200,000 (70 out of 262 or 26.7%).

Meanwhile, the percentage of respondents who answered "less than 10%" was significantly (p < 0.0001) higher in public high schools (151 out of 356, 42.4%) than in private high schools (75 out of 279, 26.9%), and significantly (p < 0.0001) higher in high schools with a population of less than 200,000 (119 out of 262 45.4%) than in schools with a population of 200,000 or more (107 out of 373 28.7%).

### (7) Number of graduates enrolled in medical school and the location of the high school

**Fig 1:**
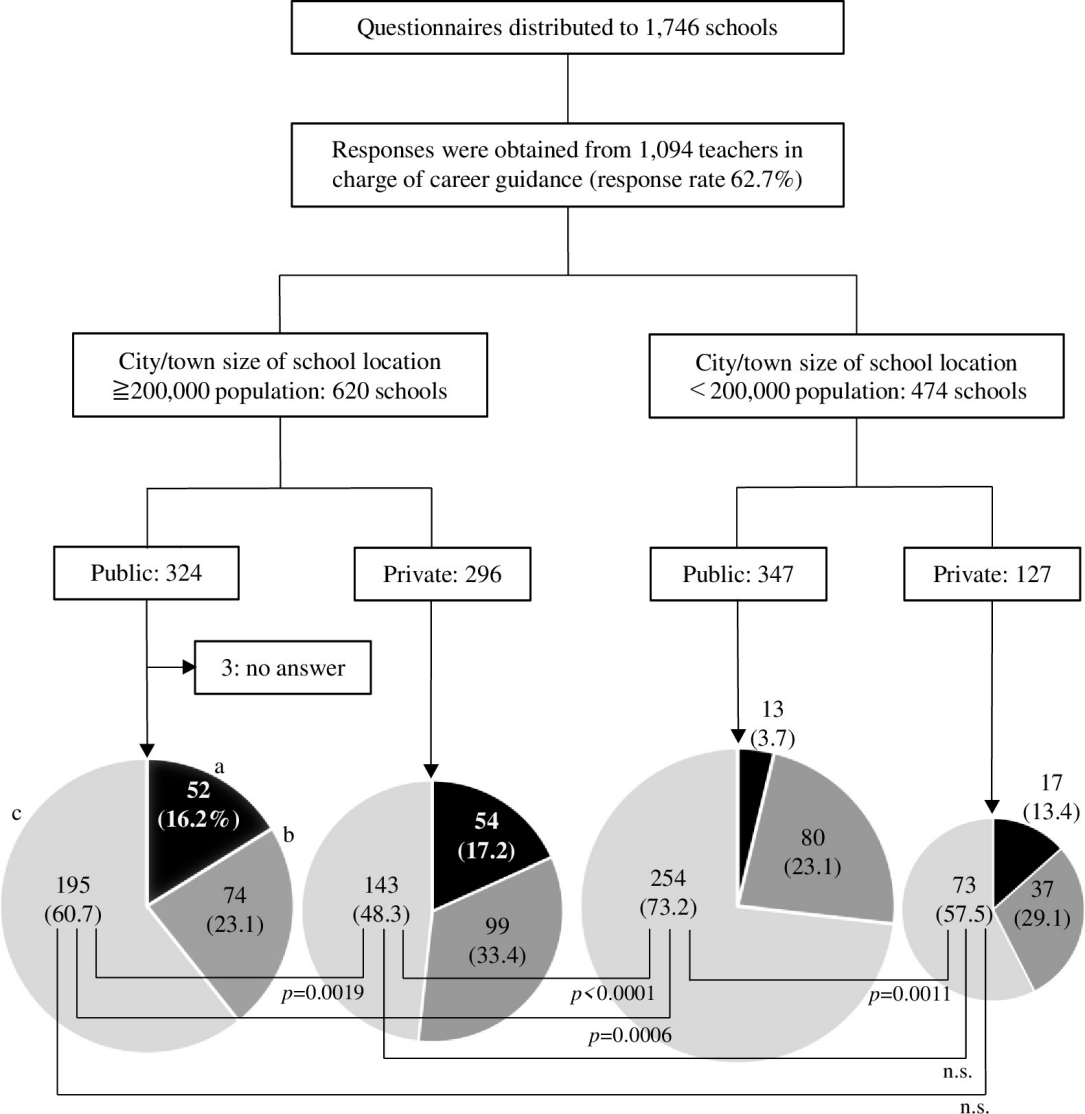
Number of students enrolled in medical school by location and the type of high school. ^a^The black portion of each pie chart shows the respondents (in numbers and percentages) who answered that the average number of students enrolled in the medical school per year was 10 or more. ^b,c^Moreover, the dark gray portion of each pie chart shows the number was 2–9, whereas the light gray portion shows the number was one or less.

Fig 1 shows a graph of the 1,091respondents to the question regarding the number of graduates enrolled in medical schools, divided according to the population size of the municipality where the high school is located and by type.

The highest percentage of high schools with two or more graduates enrolled in medical school on an annual average basis were private ones in cities with a population of 200,000 or more, 153 out of 296 schools (51.7%). Further, the lowest percentage was in municipalities with a population of less than 200,000–93 out of 347 (26.8%).

There were 106 (52 public and 54 private) schools in cities with a population of 200,000 or more, compared to 30 (13 public and 17 private schools) schools in cities, towns, and villages with less than 200,000 residents, where more than 10 students advanced to a medical school in the average year.

### (8) Percentage of parents who are doctors or dentists

Regarding the students aspiring to go to medical school, we asked about the percentage of parents who were doctors or dentists. Out of 1,068 respondents, 390 (36.5%) answered, "I do not know." 257 (24.1%), 166 (15.6%), and 255 (23.9%) answered "9% or less," "10%-49%," and "50% or more" respectively.

In private high schools, 57.2% (163 out of 285 schools; excluding the response “I don’t know” and no answer) answered "50% or more,” and in public high schools, the ratio was 23.4% (92 out of 393 schools), which was significantly lower (p < 0.001).

## Discussion and conclusions

As is widely recognized, passing the entrance examination of a medical school is the first step toward becoming a medical doctor. In this survey, about two-thirds of the respondents affirmed that it is difficult for underprivileged students to advance to medical school. It can be seen that individuals from wealthy families are more advantageously positioned in this regard.

From the survey data, the percentage of high schools with two or more graduates enrolled in a medical school on an annual average basis was higher in private schools than in public schools and schools located in large cities than in small municipalities. The percentage of respondents who admitted that some students gave up on aspiring to enter a medical school for financial reasons was particularly high in the Kanto area. Although approximately 33.8% of the total population of Japan lives in this area, the number of medical schools is rather small at 23 out of 79 (29.1%). Moreover, this result may be related to fewer public schools in this area—6 out of 50 (12%). From these results, it can be inferred that a medical school with low tuition fees will help open doors to admission for more people.

These results suggest that the biggest factor hindering aspirations for a medical career is the fact that the medical school entrance exam requires specialized preparation.

Aside from the fact that children from less wealthy families are unable to enroll in private medical schools because of their high tuition fees, it is also challenging to pass the entrance examination for a public medical school without considerable preparation. Attending a prep school that specializes in medical school entrance exams or a high school with a high medical school entrance rate can put one in an advantageous position. It can be inferred that in families with economic difficulty, candidates may give up on applying to medical schools. Additionally, even at a public medical school, the annual tuition fee alone is 535,800 yen (about 5,000 dollars), making it daunting for economically disadvantaged families to pay the amount for six years.

In our survey, about one in four respondents affirmed that the parents of more than half of the students aiming for medical school were doctors or dentists. Polyakova et al. [12] reported an increasing proportion of medical students’ parents in Sweden who are physicians. Brown called “parentocracy” the social mechanisms and norms in which the achievement of a child’s education depends on the parent’s interest in education and active support [13]. From this survey, it was not possible to examine in detail whether having parents who are physicians or dentists affects students’ aspirations to become medical doctors, but parentocracy may be prevalent in Japanese medical education.

Why is there a tendency in Japan to insist on academic achievement tests that are unique to each medical school? In this regard, Bourdieu called non-monetary educational background and cultural background “cultural capital.” He said that parents with a rich cultural capital have a favorable effect on the achievement of their children’s status [14]. Alternatively, Tachibanaki [15] argued that in Japan, the degree of trust and dependence on “learning capital” is considerably higher than that on cultural capital. Learning capital is a concept advocated by Kariya [16], who states that "learning competencies are the core of human capital." In other words, there might be a tendency in Japan to emphasize an individual’s learning efforts and the abilities obtained by them rather than their origin and environment. Many of the entrance exams of famous Japanese universities, not limited to medical schools, seem to dictate the pass or fail criteria according to the so-called academic ability principle, rather than setting the criteria based on the necessary abilities and measuring them on multiple axes.

In Japan, the birthrate has been declining, and the population is aging at one of the fastest rates in the world [17]. We need to maintain or increase the number of trained doctors and the quality of medical care provided by a small pool of young people [18]. To help students from disadvantaged backgrounds, the use of entrance exams that require overly specialized preparation must be reevaluated. Instead, the Common Test for University Admissions could be used as an initial screening to ensure the candidates’ academic ability to learn after entry to medical school. Thereafter, it is desirable to measure the candidates’ abilities according to the school’s admission policy, for instance, in terms of communication skills, sense of ethics, or altruistic values. In addition, it might be possible to give students with a disadvantaged background (by reference to resident area, location of the high school they graduated from, or school type) priority for enrollment in event that candidates show similar abilities.

This research has some limitations. First, since it was not targeted at high school students and their parents and was an indirect survey of teachers in charge of career guidance, detailed numerical values and decision-making factors regarding the background of individual high school students could not be measured. Second, the recovery rate was approximately 60%, which may not fully reflect the overall trend. Third, nearly five years have passed since the survey was conducted, and there is a concern that it does not reflect the latest situation. Fourth, our survey only covered the high schools ranked in terms of the number of students who successfully enrolled in higher education institutions, not all of the high schools. In addition, this survey was conducted only in Japan, and the results cannot be applied to other countries. However, this is one of the first nationwide surveys to explore the financial and geographical impact of medical school admission, which should be developed further to include in-depth data about the decisions and experiences of high schools, medical schools, students, and other stakeholders such as parents.

In conclusion, this survey revealed that many high school teachers in charge of career guidance recognize that students from wealthy families and living in urban areas have an advantage in advancing to medical school. The responses from high school teachers imply that some students have to abandon future studies in medicine for financial reasons. More comprehensive demographic data of medical school matriculants is needed to help clarify the influence of economic background on students’ choices and enrollment. Therefore, future research should integrate the data from high schools more extensively across the country to discuss a new design of medical school recruitment to diversify the medical student population.

## Supporting information

S1 Table Minimal underlying data set. School names and prefecture IDs of school locations have been removed to protect confidentiality of the survey participants.
(XLS)
